# Classification Modeling Method for Near-Infrared Spectroscopy of Tobacco Based on Multimodal Convolution Neural Networks

**DOI:** 10.1155/2020/9652470

**Published:** 2020-02-12

**Authors:** Lei Zhang, Xiangqian Ding, Ruichun Hou

**Affiliations:** College of Information Science and Engineering, Ocean University of China, Qingdao 266100, China

## Abstract

The origin of tobacco is the most important factor in determining the style characteristics and intrinsic quality of tobacco. There are many applications for the identification of tobacco origin by near-infrared spectroscopy. In order to improve the accuracy of the tobacco origin classification, a near-infrared spectrum (NIRS) identification method based on multimodal convolutional neural networks (CNN) was proposed, taking advantage of the strong feature extraction ability of the CNN. Firstly, the one-dimensional convolutional neural network (1-D CNN) is used to extract and combine the pattern features of one-dimensional NIRS data, and then the extracted features are used for classification. Secondly, the one-dimensional NIRS data are converted into two-dimensional spectral images, and the structure features are extracted from two-dimensional spectral images by the two-dimensional convolutional neural network (2-D CNN) method. The classification is performed by the combination of global and local training features. Finally, the influences of different network structure parameters on model identification performance are studied, and the optimal CNN models are selected and compared. The multimodal NIR-CNN identification models of tobacco origin were established by using NIRS of 5,200 tobacco samples from 10 major tobacco producing provinces in China and 3 foreign countries. The classification accuracy of 1-D CNN and 2-D CNN models was 93.15% and 93.05%, respectively, which was better than the traditional PLS-DA method. The experimental results show that the application of 1-D CNN and 2-D CNN can accurately and reliably distinguish the NIRS data, and it can be developed into a new rapid identification method of tobacco origin, which has an important promotion value.

## 1. Introduction

The origin of tobacco directly determines the difference of the style characteristics and intrinsic quality of tobacco [1] and also serves as an important basis for highlighting the characteristics of different brands of cigarettes. At present, the identification of tobacco origin mainly relies on sensory assessment by experts, genetic detection, and chemical composition detection. However, due to the complicated process, long time period, and high cost, these methods cannot be widely applied and rapidly realize identification of tobacco origin. Therefore, it is necessary to study a method that can identify the tobacco origin quickly, accurately, and conveniently.

Near-infrared spectroscopy has been widely used in the quantitative detection and qualitative analysis of tobacco fields because of its advantages of simplicity, rapidity, low cost, environmental protection, large amount of information, and simultaneous determination of multiple components [2]. Many researchers have attempted to identify tobacco origins by pattern recognition methods based on near-infrared spectrum (NIRS). For example, Shu et al. [3] used principal component analysis combined with support vector machine algorithm (PCA-SVM) to establish a NIRS origin identification model for flue-cured tobacco in six provinces of China. The partial least squares discriminant analysis algorithm (PLS-DA) was used to establish a tobacco NIRS origin identification model including four provinces in China [4]. The neural network method was used to identify the origin pattern of more than 1000 tobacco samples from the United States and abroad by near-infrared spectroscopy [5]. Most studies have shown that the direct construction of the identification model by full NIRS will not only increase the modeling complexity but also reduce the recognition performance and generalization ability, due to its high dimensionality, high-frequency noise, and redundant information. The usual practice is to use data dimensionality reduction to compress high-dimensional spectral data into low-dimensional space and improve model recognition performance while preserving more feature information as much as possible. Most researchers use the PCA method to reduce the dimensionality of high-dimensional spectral data [6, 7], but PCA is a linear algorithm, which cannot explain the complex polynomial relationship between features, and does not consider the category information of the data.

Convolutional neural network (CNN) first achieved success in the field of image recognition and has been gradually widely used in other fields. Zhang et al. [8] proposed a fault diagnosis model based on deep one-dimensional convolutional neural network (1-D CNN), and a larger size convolution kernel and a neurons dropout method were used to realize accurate and stable diagnosis based on the original vibration signal. A fault diagnosis model based on LeNet-5 network was proposed, and the original time domain signal was converted into a two-dimensional gray image as the model input to realize fault diagnosis based on one-dimensional time domain signal [9]. It is concluded that the powerful feature extraction ability of the CNN is very suitable for the pattern feature extraction of NIRS, and the extracted features are used for classification. However, at present, there are few reports on the classification of NIRS based on CNN.

In view of the above problems, this paper presents the method of multimodal CNN to identify the NIRS of tobacco from different regions. The NIR-CNN identification model of tobacco origins is established by using NIRS of 5,200 tobacco samples from 10 major tobacco producing provinces in China and 3 foreign countries. With the excellent feature learning ability of CNN, the reconstructed feature data can better reflect the essential features of NIRS, which is helpful for the classification of spectral data. Firstly, simple patterns in one-dimensional near-infrared spectrum (1-D NIRS) are identified by the lower layer of 1-D CNN, then these simple patterns are combined into complex features in the upper layer, and then the extracted features are used for classification. Secondly, the 1-D NIRS are converted into two-dimensional spectral images, and the structure features are extracted from two-dimensional spectral images by the image convolution method. The global training features are extracted and classified by mining the spatial correlation in the data. Finally, the optimal CNN model is selected by adjusting the network structure parameters, and the identification performances of different spectral processing methods and different models are compared.

## 2. Materials and Methods

### 2.1. Sample Preparation

Data balancing is very important in multiclassification problems. Generally, the data in the training set should be distributed as evenly as possible with respect to the category labels, that is to say, the data sets corresponding to each category label are basically equal in the training set, so as to avoid the classification model being too inclined to the characteristics of some certain categories. In this research work, a total of 5,200 tobacco samples were collected from 10 major tobacco planting provinces of China (Yunnan, Guizhou, Fujian, Jiangxi, Hunan, Sichuan, Chongqing, Henan, Shandong, and Jilin) and 3 major foreign countries (Brazil, Zimbabwe, and America). That means 400 tobacco samples were taken from each province or country. Among them, 320 samples were used for training and 80 samples were used for testing. The origins and quantities of all observations are summarized in Table 1. All origins of tobacco samples were identified by experts from State Tobacco Monopoly Administration of China.

### 2.2. Near-Infrared Spectra

NIRS of tobacco samples were acquired by Antaris-II with diffuse reflection mode, which is a Fourier-transform near-infrared spectrometer produced by ThermoFisher, America. The spectral scanning range was 4000 cm^−1^∼10000 cm^−1^, the resolution was 8 cm^−1^, and the number of scans was 64. Each sample of tobacco was dried in an oven (60°C, 2 h) and grounded into powder (60 meshes). In order to ensure the consistency of spectral acquisition, each sample was repeatedly scanned for three times, and the mean spectrum was used as the final spectrum of the sample.

### 2.3. Spectral Processing

Spectral data processing and algorithm calculation were based on MATLAB, version 2016b. Due to the problems, such as baseline drift, high-frequency noise, and mutual interference between components, in the raw NIRS, it is necessary to perform corresponding preprocessing before applying the spectra to improve the signal-to-noise ratio and enhance the prediction performance of the model. In this work, the first-order derivative and SavitzkyGolay (S-G) smoothing methods were adopted, the moving window width was 9, and the polynomial number was 3 [10].

**Figure 1:**
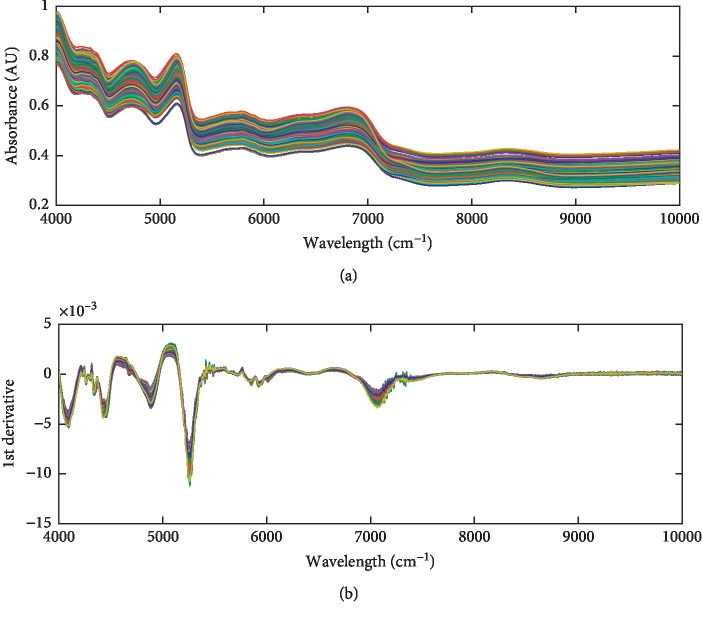
The raw NIRS of the tobacco samples (a) and the 1st derivative and S-G smoothing preprocessed NIRS (b).

Figure 1(a) shows the raw NIRS of the tobacco samples collected by the method mentioned above.  Figure 1(b) shows the processed NIRS which are calculated after the 1st derivative and S-G smoothing. It can be seen from Figure 1 that the 1st derivative and smoothing processes effectively reduce the baseline drift and highlight the differences in the raw spectra of different samples, which is very important for the qualitative classification of the NIRS.

### 2.4. Convolutional Neural Network (CNN)

CNN is a machine learning model under deep supervised learning, which requires very little data preprocessing and is highly adaptable [11]. By forward propagation through the filters of each layer in the network, the local features of the data can be better mined, and the global training features can be extracted and classified. Its weight-sharing structure network makes it more similar to biological neural networks and has achieved good results in various fields of pattern recognition.

The basic structure of the CNN consists of an input layer, a convolution layer, a pooling layer, a fully connected layer, and an output layer [12]. Each feature map has a plurality of neurons and extracts a feature of the input through a convolution filter. The convolution layer and the pooling layer are the core modules for implementing the feature extraction function of the CNN. The lower hidden layer of the CNN is composed of the convolution layer and the pooling layer alternately. The upper layer is the fully connected layer corresponding to the hidden layer and regression classifier of the traditional multilayer perceptron (MLP) [13]. Various classifiers can be used to classify the feature data extracted from the lower layer. The network model minimizes the loss function by using the gradient descent method to reversely adjust the weight parameters in the network layer by layer [14] and improves the accuracy of the network through frequent iterative training.

#### 2.4.1. Convolutional Layer Operation

**Figure 2:**
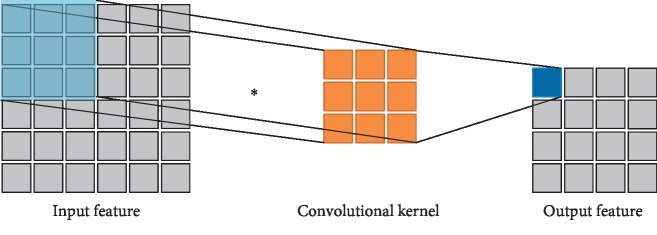
Schematic diagram of the two-dimensional convolution operation.

The convolutional layer convolves the input data using a plurality of convolutional kernels and outputs the convolved features [15], that is, the feature maps. Each convolutional kernel outputs a feature map corresponding to a type of extracted features. Since it is not affected by the input dimensions and is easy to train the depth structures, the convolution structure is an effective tool for feature extraction of complex and high-dimensional inputs. The two-dimensional convolution operation process is shown in Figure 2.

The input data and the convolution kernel are both two-dimensional tensors. The convolution calculation formula is as follows:

(1) $$\begin{array}{c} y\left(i\right)=f\left(g\left(i\right)\right)=f\left(\sum_{x=1}^{m}\sum_{y=1}^{n}a_{x,y}*w_{x,y}^{i}+b^{i}\right), \end{array}$$

where *i* is the *i*-th convolution kernel; *g*(*i*) is the feature map of the *i*-th convolution kernel; *a* is the input data; *b* is the bias; *x* and *y* are the two dimensions of the input data; and *f*(·) is an activation function used to implement nonlinear transformations.

The ReLU activation function is the most widely used in CNN at the present stage [16], and its calculation process is as follows:

(2) $$\begin{array}{c} f\left(g\left(i\right)\right)=\max\left\{0,g\left(i\right)\right\}. \end{array}$$

#### 2.4.2. Pooling Layer Operation

The pooling layer downsamples the input feature vectors by the sampling kernels and further highlights the extracted features while realizing data dimensionality reduction [17]. The pooling operation mainly includes the maximum pooling and the average pooling [18], as shown in the following equation:

(3) $$\begin{array}{c} p_{\max}^{l\left(i,j\right)}=\underset{\left(j-1\right)w<t<jw}{\max}\left\{a^{l\left(i,t\right)}\right\},\\ p_{\text{avg}}^{l\left(i,j\right)}=\underset{\left(j-1\right)w<t<jw}{\text{avg}}\left\{a^{l\left(i,t\right)}\right\}, \end{array}$$

where *a*^*l*(*i*, *t*)^ is the *t*-th neuron of the *i*-th feature map in the *l*-th layer, *w* is the width of the convolution kernel, and *j* is the *j*-th sampling kernel.

### 2.5. One-Dimensional Convolutional Neural Network (1-D CNN)

**Figure 3:**
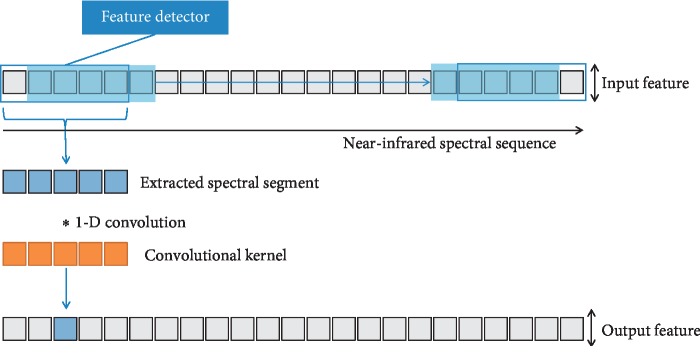
Schematic diagram of one-dimensional convolution operation.

1-D CNN is a special CNN that can be well applied to one-dimensional time series analysis. It also can be used to analyze signal data with fixed length period or fixed position expression, such as NIRS data. The input of 1-D CNN is a one-dimensional vector, so the convolution kernel and feature map of the network are also one-dimensional. As with the two-dimensional CNN, the same pooling operation can be performed for the one-dimensional vectors, which is also used to extract the data features of the one-dimensional vectors. The one-dimensional convolution operation process is shown in Figure 3.

The convolution calculation process is shown in

(4) $$\begin{array}{c} y\left(i\right)=f\left(g\left(i\right)\right)=f\left(\sum_{x=1}^{m}a_{x}*w_{x}^{i}+b^{i}\right), \end{array}$$

where *i* is the *i*-th convolution kernel; *g*(*i*) is the feature map of the *i*-th convolution kernel; *a* is the input data, *b* is the bias; *x* is the one-dimensional input data; and *f*(·) is the ReLU activation function used to implement nonlinear transformations.

### 2.6. Softmax Function

Softmax function is a gradient logarithmic normalization of a finite discrete probability distribution, which is commonly used in multiclassification processes [19]. It compresses a *K*-dimensional vector *V* with any real number to another *K*-dimensional real vector *σ*(*V*), so that every element is between 0 and 1, and the sum of all the elements is 1. The score value of each category is regarded as a probability to be understood, and the multiclassification is performed by selecting the node with the largest probability (that is, the node with the largest value) as the prediction target.

Suppose there is an array *V*, *v*_*i*_ represents the *i*-th element in *V*, then the softmax value of this element is calculated as shown in

(5) $$\begin{array}{c} S_{i}=\frac{e^{v_{i}}}{\sum_{j=1}^{n}e^{v_{j}}}, \end{array}$$

where *n* is the number of elements in the array *V*.

The softmax function is widely used in probability-based multiclassification problems. In the deep neural network, the input of softmax is the result obtained from *K* different neurons, and the probability that the sample vector *x* belongs to the *j*-th category is shown in

(6) $$\begin{array}{c} P\left(y=\left.j\right|x\right)=\frac{e^{x^{T}w_{j}}}{\sum_{k=1}^{K}e^{x^{T}w_{k}}}. \end{array}$$

It can be seen from the formula that the probability belonging to a certain class is a composite of *K* linear function softmax. There is a significant correlation between the prediction results and the input features. The more the features of a certain class are included, the higher the probability of the output being of a certain class. When several features are often activated together, the training process will learn the larger joint distribution weights, making their joint probability closer to the real category.

### 2.7. Model Training Parameters Setting

For the training of CNN, the learning rate is set to 0.01, the batch size is set to 40, and the epoch of training is set to 50. The model may appear overfitting during the training process, which can be dealt with by adjusting the model operation mode. Therefore, this paper sets early termination for CNN training. In most cases, the model first learns the correct distribution of the data and then begins to overfit the data at some point in time. As the number of iterations increases, the training error will gradually decrease, while the testing error will decrease first and then increase. When the testing error increases, it indicates that the model is overfitting. Therefore, by identifying when the testing error of the model has changed (turned up), the training process of the model can be terminated before the overfitting occurs.

### 2.8. Model Performance Evaluation Method

The performance of the model is evaluated by the data set classification accuracy index. The classification accuracy rate *P*_*A*_ is calculated as shown in

(7) $$\begin{array}{c} P_{A}=\frac{N_{C}}{N_{T}}\times 100\%, \end{array}$$

where *N*_*C*_ is the number of correctly classified samples in the data set and *N*_*T*_ is the total number of samples in the data set.

## 3. Results and Discussion

### 3.1. Research on Dimensionality Reduction Classification of NIRS

According to the 1st derivative and S-G smoothing preprocessed NIRS described in the experimental part, PCA and t-distributed stochastic neighbor embedding (t-SNE) [20] were used to reduce the dimension of the NIRS. And, the classification differentiation was preliminarily explored through the two-dimensional visual graphics.

**Figure 4:**
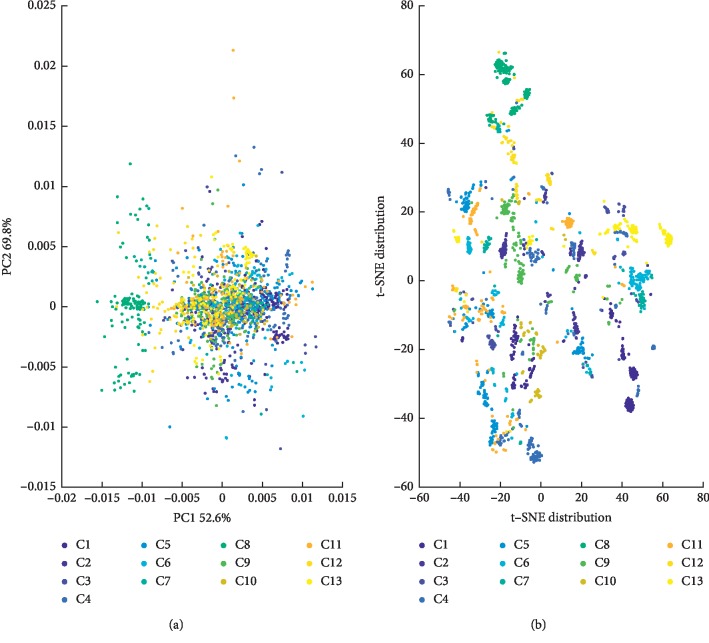
Two-dimensional feature visualization of NIRS. (a) PC1 and PC2 score maps of PCA; (b) two-dimensional feature distribution map of t-SNE.

Figure 4(a) is the PC1 and PC2 score maps of PCA. As can be seen from the figure, in the PCA model constructed from the overall samples, the contribution of the first principal component (PC1) was 52.6%, and that of the second principal component (PC2) was 17.2%. However, tobacco samples from different regions exhibited high overlap due to the same compositional properties. Therefore, the first two principal components, while characterizing the major part of the NIRS differences, are not sufficient to achieve the classification distinction.

Figure 4(b) is the 2-dimensional feature distribution map of the t-SNE algorithm. In order to further demonstrate the difference of NIRS characteristics of different sample types, the t-SNE dimensionality reduction algorithm in manifold learning was introduced to visualize low-dimensional features. It can be seen from the figure that the distribution of the class features extracted by the t-SNE algorithm is slightly better than that of the PCA. However, due to the influences of spectral redundancy, noise, and other factors, different categories are still difficult to distinguish.

This result shows that it is very difficult to classify tobacco origins by full NIRS using conventional methods. It is necessary to use a deep feature extraction method to find local and global features with better classification discrimination from the NIRS.

### 3.2. Classification Performance of 1-D CNN

**Figure 5:**
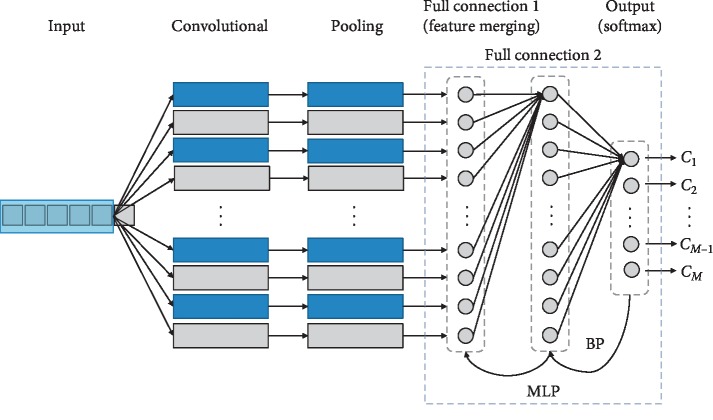
Basic structure of 1-D CNN.

In this paper, different parameters and depths of the network structure were designed for the application of 1-D CNN in the NIRS classification, and the 1-D CNN models with different network structures were applied to the tobacco origin classification. The dimension of 1-D NIRS data is 1609, so the input data value of 1-D CNN is 1 ∗ 1609. The architecture of 1-D CNN is similar to that of 2-D CNN in the field of computer vision. It consists of an input layer, a fully connected layer, an output layer, and multiple convolutional and pooling layers stacked together. Generally speaking, the depth calculation of neural network does not include the input layer, only the hidden layer, and the output layer. For the 1D-CNN designed in this paper, its network depth is 2 fully connected layers (the first fully connected layer is the feature merge layer, which summarizes the local features of all feature maps after convolution and pooling), 1 output layer, and several convolutional and pooling layers added together. For example, a 1-D CNN with a depth of 5 includes 1 convolutional layer, 1 pooling layer, 2 fully connected layers, and and 1 output layer; a 1-D CNN with a depth of 7 includes 2 convolutional layers, 2 pooling layers, 2 fully connected layers, and 1 output layer. The rest of the depths are deduced by analogy. The basic structure of 1-D CNN is shown in Figure 5.

After feature extraction through the convolutional layers and pooling layers, all feature maps are stitched and merged by the first fully connected layer to summarize all local features. The number of neural nodes in the first fully connected layer changes with the convolution kernel size, the sampling kernel size, and the number of feature maps. The number of neural nodes in the second fully connected layer is the same as that in the first fully connected layer. The last output layer is classified by softmax, and the output category is 13 in the data set. The 2 fully connected layers and the softmax layer form a simple MLP structure. Each neuron in the fully connected layer is fully connected to all neurons in the previous layer. The excitation function of each neuron in the fully connected layer adopts the ReLU function, and the training method uses the BP algorithm. In the fully connected layer, a regularization method, dropout technology, is adopted to make the output value of the neurons in the fully connected layer become 0 with a probability of 0.5. By this method, parts of the neural nodes become invalid. These nodes will neither participate in the forward propagation process of CNN nor will participate in the backpropagation process. At present, most researches on CNN use ReLU + dropout technology, which has achieved good classification performance.

Firstly, the effects of network depth on classification performance are studied by using 5 different depth network structures: (1) depth 5 (1 convolutional layer and 1 pooling layer); (2) depth 7 (2 convolutional layers and 2 pooling layers); (3) depth 9 (3 convolutional layers and 3 pooling layers); (4) depth 11 (4 convolutional layers and 4 pooling layers); (5) depth 13 (5 convolutional layers and 5 pooling layers). The simple structures of 1-D CNN at different depths are shown in Table 2, where *C* represents the convolutional layer and S represents the pooling layer. Stage represents the stacking stage of 1 convolutional layer and 1 pooling layer, and *M* represents the number of feature maps (i.e., the number of convolution kernels) of each stage.

Table 3 shows the results of the NIRS classification by the 1D-CNN models with 5 different depths on the training set and test set. In the stacked structure of each convolutional layer and pooling layer, the convolution kernel size in the convolutional layer is 1 ∗ 5, the sampling kernel size of the pooling layer is 1 ∗ 2, and the number of feature maps in each stage is 12. As can be seen from Table 3, with the increase of neural network depth, the classification accuracy of training set increases continuously, and the classification accuracy of test set first rises and then declines [21]. This shows that, as the complexity of the network structure increases, the model overfitted the training set samples, resulting in a decline in generalization ability. Therefore, the model with a depth of 11, the best comprehensive performance of training set and test set, is adopted in this paper for subsequent experiments.

#### 3.2.1. Influence of Convolution Kernel Size on Model Classification Performance

**Figure 6:**
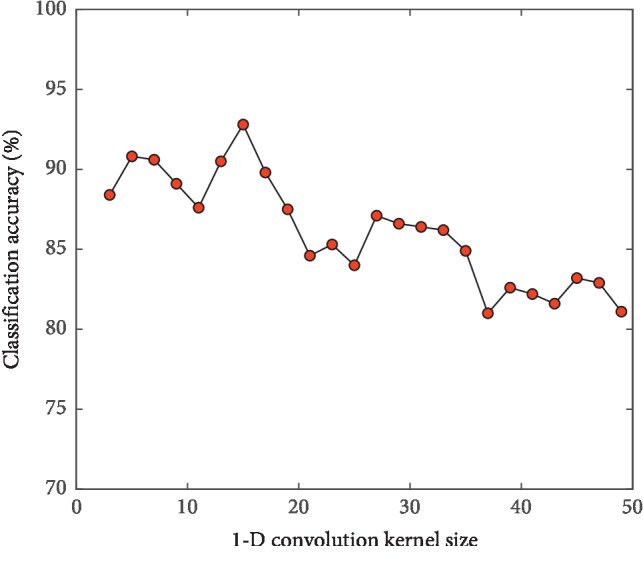
Model classification accuracy of different convolution kernel sizes.

In order to test the influence of convolution kernel size on the performance of the model, under the condition of network depth 11, the convolution kernel size is gradually increased by Step 2 from 3, and the 1D-CNN models are established when other parameters are the same. The curve of classification accuracy of the model test set changing with the size of convolution kernel is shown in Figure 6. It is found through experiments that too large or too small convolution kernel is not conducive to model learning [22]. In this experiment, when the convolution kernel size is 15, the model can obtain better classification results.

#### 3.2.2. Influence of Sampling Kernel Size on Model Classification Performance

In order to test the influence of sampling kernel size on the performance of the model, under the condition of network depth 11, the sampling kernel size is changed and the 1-D CNN models are established when other parameters are the same. The pooling layer reduces the number of neurons through the downsampling operation, so the size of sampling kernel is generally required to be smaller than the convolution kernel and capable of divisible postconvolution sequence. In this experiment, when the convolution kernel size is 1 ∗ 15, the results of classification accuracy for different sampling kernel sizes are shown in Table 4. It is found through experiments that, when the size of sampling kernel is 2, the model can obtain better classification results.

#### 3.2.3. Influence of the Number of Feature Maps on Model Classification Performance

**Figure 7:**
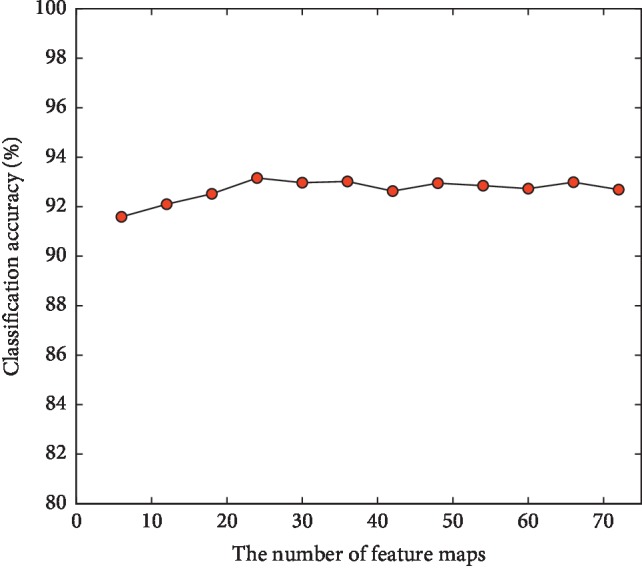
Model classification accuracy with different number of feature maps.

In order to test the influence of the number of feature maps (convolution kernels) on the performance of the model, under the condition of network depth 11, the number of feature maps is gradually increased by step 6 from 6 and the 1D-CNN models are established when other parameters are the same. The curve of classification accuracy of the model test set changing with the number of feature maps is shown in Figure 7. Through experiments, the classification accuracy increases first and then decreases. When the number of feature maps is too small, some features conducive to network learning are ignored, so its classification performance is poor [23]. However, when the number of feature maps is too large, not only the training time of the model is greatly increased but also the model is prone to overfitting. Considering comprehensively, the number of feature maps is set to 24.

In order to explore the optimal combination of CNN depth, convolution kernel size, sampling kernel size, and the number of feature maps, more than 200 CNN models with different structures are designed in this paper. These different CNN models all use the same training set and test set as above to carry out experiments.

### 3.3.  Classification Performance of  2-D CNN

In this paper, the LeNet-5 open source network [24], which is widely used in the field of two-dimensional image recognition and has a high classification accuracy, is adopted for training of two-dimensional near-infrared spectral (2-D NIRS) images. Small random numbers are used to initialize the weights of LeNet-5 network, 2-D NIRS data of the training set are used for model training, and the weights of the network are updated through back propagation. And, the last fully connected layer is replaced to create output units of origin category in the data set. The retrained CNN is used to extract the features of 2-D NIRS images, and then the extracted features are used as input to train a brand new single-layer full-connected classifier to deal with the origin classification problem of NIRS.

In the case of a fixed CNN structure, the image structure of 2-D NIRS becomes an important factor affecting the classification accuracy. In order to test the influence of 2-D NIRS image structure on the classification performance of the model, the 2-D CNN models are established under the condition that other parameters are the same by changing the input structure of 2-D NIRS images combined with the extracted characteristic spectrum segments of the NIRS. The NIRS with different spectral lengths are transformed into 2-D NIRS images as follows.

**Figure 8:**
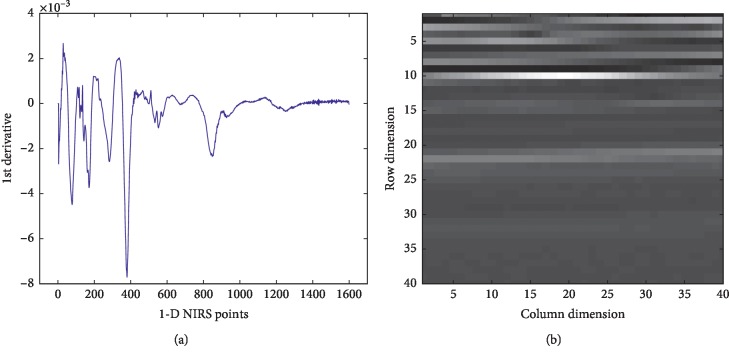
Process of converting 1-D NIRS to 2-D NIRS. (a) 1-D NIRS; (b) 2-D NIRS.

1-D NIRS data are a series of digital vectors. When converting it into a 2-D image matrix, it is necessary to intercept subvectors of a fixed length from front to back and stack the subvectors in rows.  Figure 8(a) illustrates a process of converting 1-D NIRS to 2-D NIRS by taking a one-dimensional near-infrared spectrum at 1600 points as an example. Since 1600 can be decomposed into 40 ∗ 40, the converted 2-D NIRS image has 40 dimensions for both rows and columns. Take the values of points 1–40 in 1-D NIRS and put them into the first row of the two-dimensional matrix. Take the values of points 41–80 in 1-D NIRS and put them into the second row of the two-dimensional matrix. By analogy, a 2-D NIRS image of 40 ∗ 40 can be formed, as shown in Figure 8(b).

**Figure 9:**
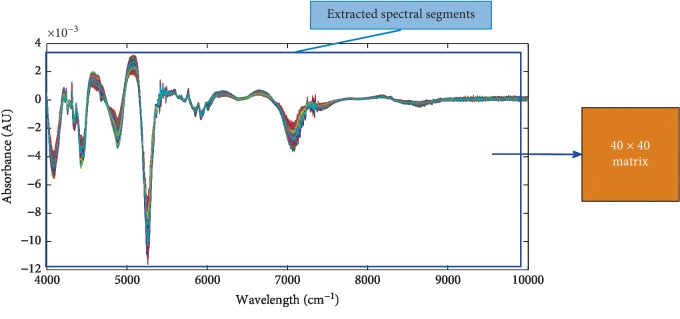
Full NIRS transformed into a two-dimensional spectral image.

Full spectrum conversion is shown in Figure 9. 1-D NIRS data have 1609 detection points. After removing the last 9 points, it becomes 1 ∗ 1600, which is converted into a two-dimensional image matrix of 40 ∗ 40.

**Figure 10:**
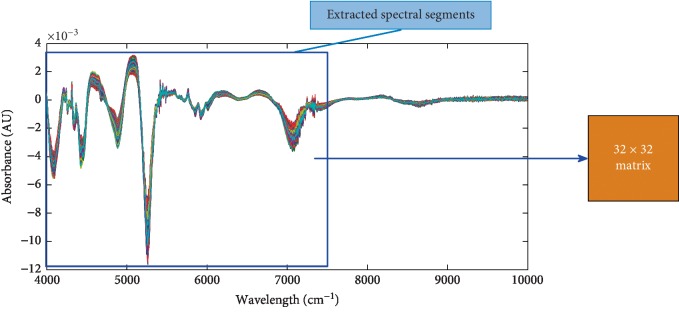
Feature NIRS transformed into a two-dimensional spectral image.

Feature segment spectrum conversion is shown in Figure 10. For the spectrum range of “4000 cm^−1^∼7740 cm^−1^” with high signal-to-noise ratio, there are 1024 detection points in 1-D NIRS, which is converted into a two-dimensional image matrix of 32 ∗ 32.

The NIRS is gradually intercepted from the right side by changing the width of the window, and the 2-D NIRS image size is compressed in a step of 2.  Table 5 shows the classification accuracy of different 2-D spectral image sizes from 40 ∗ 40 to 26 ∗ 26 on the test set.

Through the experiments, the accuracy of model classification increases first and then decreases. When the 2-D NIRS image size is 32 ∗ 32, the classification accuracy of the model reaches the highest level. This is because the effective signal is weak in the spectrum range of “7740 cm^−1^∼10000 cm^−1^”, mainly the noise information. After filtering out this part of the data, it can effectively highlight the effective information of the spectrum. When the spectral compression range is large, for example, the spectrum range is “4000 cm^−1^∼6400 cm^−1^”, the loss of effective spectral information leads to insufficient training of the model. Therefore, it is necessary to extract a suitable feature segment for two-dimensional spectral image conversion.

### 3.4. Classification Performance Comparison of Different Models

The best performing classification models of 1-D CNN and 2-D CNN were selected by experiments, and the same training set and test set were used for modeling analysis. Then, they were compared with the PLS-DA classification method. The classification performance of different models is shown in Table 6. The overall prediction performances of 1-D CNN and 2-D CNN are both better than those of PLS-DA.

**Figure 11:**
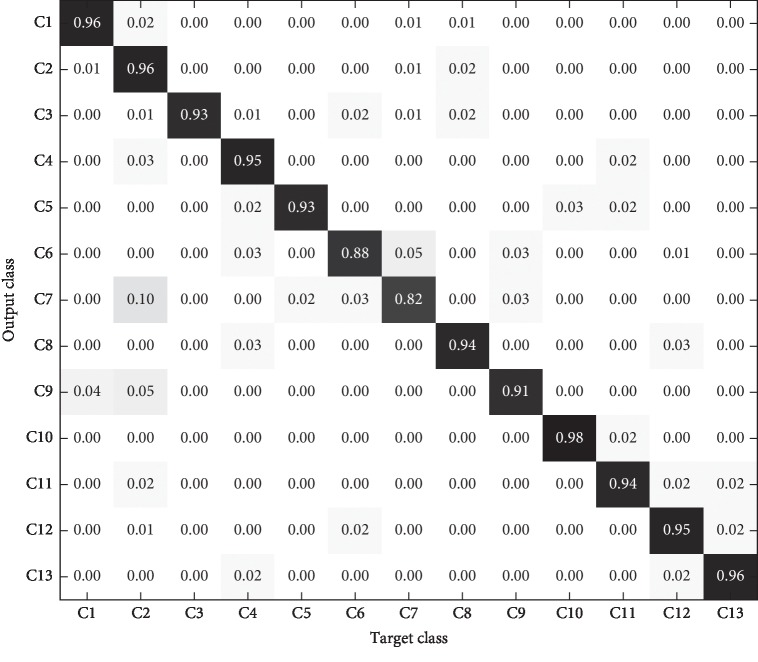
1-D CNN model identification results of test set.

**Figure 12:**
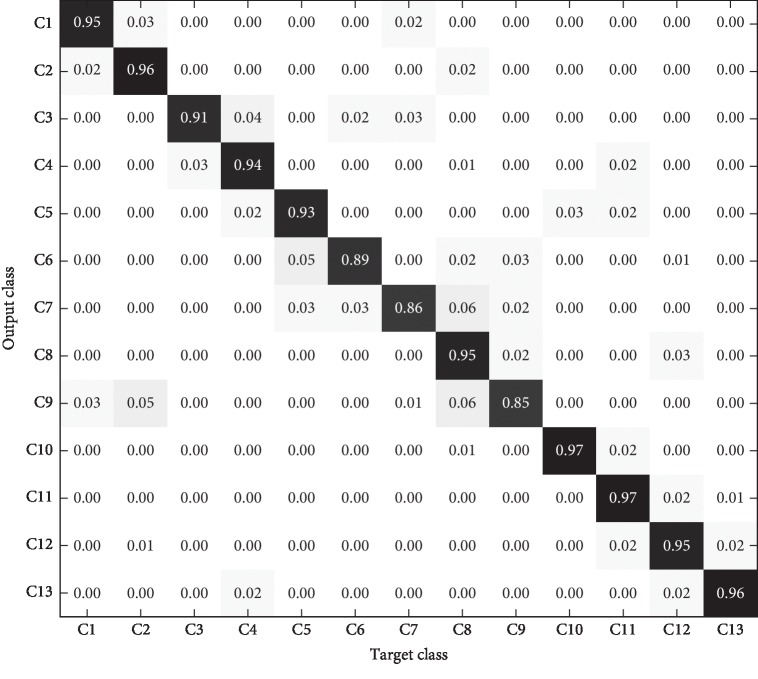
2-D CNN model identification results of test set.

**Figure 13:**
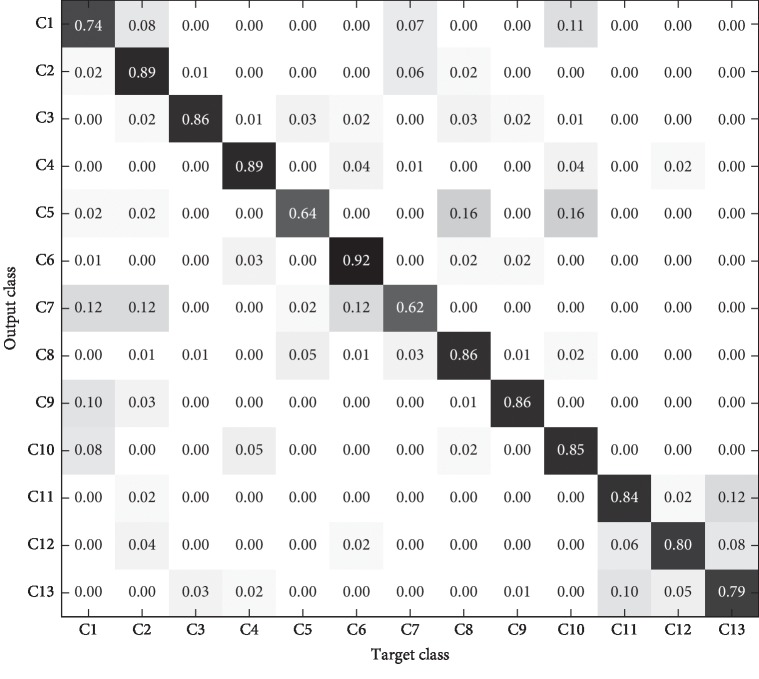
PLS-DA model identification results of test set.

In order to more clearly show the recognition results of different models for each category in the test set, the confusion matrix is introduced to analyze the experimental results in detail. The confusion matrixes of the three models are shown in Figures 11–13.

As can be seen from the comparison of the confusion matrix in Figures 11–13, the following can be observed. (1) The prediction accuracy of 1-D CNN and 2-D CNN models for each category is higher than that of the PLS-DA model, which can basically meet the requirements of practical application. (2) For some categories, the prediction accuracy of the three models is low (for example, C7), which may be caused by the lack of significant characteristics in the samples of this category. (3) The category distributions of 1-D CNN and 2-D CNN models are relatively concentrated, while the category distribution of PLS-DA model is relatively discrete.

## 4. Conclusions

In this paper, for the problem of identifying tobacco origin by NIRS, a multimodal CNN method is proposed to construct classification models for improving classification accuracy. With the current popular research method of deep CNN, NIRS data are directly input into the network. The CNN can directly extract feature information from the spectrum and conduct automatic learning, thus avoiding the accumulation of errors caused by manual feature extraction and finally achieving the accurate classification of tobacco origin.

The 1-D CNN and 2-D CNN models have achieved good classification results in most tobacco origin categories, and both of the model performances are better than the traditional PLS-DA model. This method can be developed into an accurate and high performance method for identifying tobacco origin, which has important guiding significance and extensive popularization value for the scientific and rational utilization of tobacco raw materials of brand characteristic cigarettes.

In this paper, only some CNNs with specific structures are considered. How to adaptively select the network structures in the face of complex and variable input data remains to be further studied. In addition, when using CNN for NIRS classification, the input dimension should be consistent. Therefore, the original data need to be intercepted to a fixed length. How to intercept the appropriate data so that CNN can maximize its advantages is a problem worthy of further study. Since recurrent neural network (RNN) can handle data of varying lengths [25], it is possible to consider combining RNN with CNN in the later stage.

## Figures and Tables

**Table 1:** The origin and quantity of different tobacco samples.

Origins of tobacco samples (country/province)		Category tag	Number of training set samples	Number of test set samples
China	Yunnan	C 1	320	80
	Guizhou	C 2	320	80
	Fujian	C 3	320	80
	Jiangxi	C 4	320	80
	Hunan	C 5	320	80
	Sichuan	C 6	320	80
	Chongqing	C 7	320	80
	Henan	C 8	320	80
	Shandong	C 9	320	80
	Jilin	C 10	320	80
	Brazil	C 11	320	80
	Zimbabwe	C 12	320	80
	America	C 13	320	80

**Table 2:** Simple 1-D CNN structures of different depths.

Model	Hidden layer structure	Feature map structure	Depth
1D_CNN_5	Stage1: C1 + S1	M1	5

1D_CNN_7	Stage1: C1 + S1; Stage2: C2 + S2	(M1, M2)	7

1D_CNN_9	Stage1: C1 + S1; Stage2: C2 + S2; Stage3: C3 + S3	(M1, M2, M3)	9

1D_CNN_11	Stage1: C1 + S1; Stage2: C2 + S2; Stage3: C3 + S3; Stage4: C4 + S4	(M1, M2, M3, M4)	11

1D_CNN_13	Stage1: C1 + S1; Stage2: C2 + S2; Stage3: C3 + S3; Stage4: C4 + S4; Stage5: C5 + S5	(M1, M2, M3, M4, M5)	13

**Table 3:** Classification performance of 1-D CNN models with different depths.

Model	Depth	Training set classification accuracy (%)	Test set classification accuracy (%)
1D_CNN_5	5	82.75	74.71
1D_CNN_7	7	88.1	81.28
1D_CNN_9	9	92.46	87.05
1D_CNN_11	11	95.56	90.81
1D_CNN_13	13	96.72	88.13

**Table 4:** Classification performance of 1-D CNN models with different sampling kernel sizes.

Model	Convolution kernel size	Sampling kernel size	Test set classification accuracy (%)
1D_CNN_11	1 ∗ 15	1 ∗ 1	91.14
	1 ∗ 15	1 ∗ 2	92.82
	1 ∗ 15	1 ∗ 3	90.05
	1 ∗ 15	1 ∗ 4	89.53
	1 ∗ 15	1 ∗ 5	88.47

**Table 5:** Model classification accuracy with different 2-D image input sizes.

Model	2-D NIRS image sizes	Feature spectrum range of 1-D NIRS (approximate)	Test set classification accuracy (%)
LeNet-5	40 ∗ 40	4000–10000 cm^−1^	81.67
	38 ∗ 38	4000–9360 cm^−1^	87.56
	36 ∗ 36	4000–8800 cm^−1^	90.81
	34 ∗ 34	4000–8250 cm^−1^	92.47
	32 ∗ 32	4000–7740 cm^−1^	93.05
	30 ∗ 30	4000–7260 cm^−1^	91.83
	28 ∗ 28	4000–6810 cm^−1^	84.46
	26 ∗ 26	4000–6400 cm^−1^	78.72

**Table 6:** Classification performance of different models.

Model	Main parameters of model	Test set classification accuracy (%)
1D_CNN_11	C: 1 ∗ 15; S: 1 ∗ 2;*M* = 24	93.15
2D_LeNet-5	Input size: 32 ∗ 32	93.05
PLS_DA	Characteristic spectrum: 4000 cm^−1^∼7740 cm^−1^; PC number: 7	81.25

## Data Availability

The NIRS data of tobacco used to support the findings of this study are available from the corresponding author upon request because these data are obtained through relevant projects which can only be used for scientific research and cannot be used for any commercial purposes.
